# Reducing Alcohol and Opioid Use Among Youth in Rural Counties: An Innovative Training Protocol for Primary Health Care Providers and School Personnel

**DOI:** 10.2196/21015

**Published:** 2020-11-06

**Authors:** Erica Francis, Kara Shifler Bowers, Glenn Buchberger, Sheryl Ryan, William Milchak, Jennifer Kraschnewski

**Affiliations:** 1 College of Medicine Penn State University Hershey, PA United States; 2 Penn State Health Hershey, PA United States

**Keywords:** alcoholism, adolescent behavior, binge drinking, rural health, underage drinking, adolescent, young adult, alcohol, drinking, behavior, screening, intervention, referral

## Abstract

**Background:**

Given that youth alcohol use is more common in rural communities, such communities can play a key role in preventing alcohol use among adolescents. Guidelines recommend primary care providers incorporate screening, brief intervention, and referral to treatment (SBIRT) into routine care.

**Objective:**

The aim is to train primary care providers and school nurses within a rural 10-county catchment area in Pennsylvania to use SBIRT and facilitate collaboration with community organizations to better coordinate substance use prevention efforts.

**Methods:**

To build capacity to address underage drinking and opioid use among youth aged 9-20 years, this project uses telehealth, specifically Project ECHO (Extension for Community Healthcare Outcomes), to train primary care providers and school nurses to address substance use with SBIRT. Our project will provide 120 primary care providers and allied health professionals as well as 20 school nurses with SBIRT training. Community-based providers will participate in weekly virtual ECHO sessions with a multidisciplinary team from Penn State College of Medicine that will provide SBIRT training and facilitate case discussions among participants.

**Results:**

To date, we have launched one SBIRT ECHO project with school personnel, enrolling 34 participants. ECHO participants are from both rural (n=17) and urban (n=17) counties and include school nurses (n=15), school counselors (n=8), teachers (n=5), administrators (n=3), and social workers (n=3). Before the study began, only 2/13 (15.5%) of schools were screening for alcohol use.

**Conclusions:**

This project teaches primary care clinics and schools to use SBIRT to prevent the onset and reduce the progression of substance use disorders, reduce problems associated with substance use disorders, and strengthen communities’ prevention capacity. Ours is an innovative model to improve rural adolescent health by reducing alcohol and opioid use.

**International Registered Report Identifier (IRRID):**

DERR1-10.2196/21015

## Introduction

### Background

One in three Pennsylvanian eighth-graders has used alcohol, a substantially greater proportion than the national average (23%) [1]. By the 12th grade, nearly 70% of Pennsylvanian youth have used alcohol, compared to 60% nationally [1]. The use of alcohol and other substances by youth has significant negative public health effects, given that youth are more susceptible to risk-related injuries [2]. For example, substance use by youth correlates with increased sexual risk-taking and, due to neurodevelopmental vulnerabilities of the adolescent brain, increased risk of addiction [3,4]. In adolescents, 75% of deaths are the result of unintentional injury, homicide, or suicide, and alcohol is involved in more than one-third of adolescent deaths [5]. The Substance Abuse and Mental Health Services Administration (SAMHSA) has estimated that fewer than 10% of adolescents in need of specialty substance abuse treatment receive it [6]. Together, these factors make addressing underage drinking one of the nation’s top substance abuse prevention priorities.

### Adolescent Alcohol and Opioid Use

Youth who live in rural communities use alcohol at greater rates than their urban counterparts (37.8% vs 34.3%), highlighting the importance of intervention directed at rural youth [6]. Higher rates of alcohol use among rural youth are attributed to less parental disapproval surrounding use, greater acceptability among peers, greater availability of alcohol, and greater access to alcohol provided by adults [7]. Despite the higher prevalence, rural communities face major barriers to screening and treatment for substance use disorders, including limited resources, fewer providers and facilities, and the sparse distribution of services. In Pennsylvania, there are 48 rural counties and 19 urban counties. Furthermore, 235 of the state’s 500 public school districts are considered rural due to the student and town population density and location of student homes, even though some of these districts are in urban counties [8].

In addition to addressing alcohol use, this protocol proposes addressing opioid use, given the screening, brief intervention, and referral to treatment (SBIRT) approach’s unique ability to address substance use more broadly. Pennsylvania has the third-highest rate of deaths due to drug overdose in the country (37.9 per 100,000), and this rate has significantly increased in recent years [9]. The SBIRT model is particularly appropriate for addressing opioid use in teens, and the screening tools developed for identifying underage alcohol use have also been validated for identifying opioid use. These include the CRAFFT, S2BI, BSTAD, and 2-question NIAAA screener. Implementing broad substance use prevention strategies within clinics, schools, and community coalitions may help to drive population-level change through a collaborative approach.

### Reducing Adolescent Alcohol Consumption

One gap in efforts to reduce underage drinking is the lack of appropriate screening, intervention, and referral for alcohol use among youth. Guidelines recommend that primary care providers incorporate SBIRT into routine care [10]. SBIRT has been defined by SAMHSA as “a comprehensive integrated public health approach to the delivery of intervention for individuals with risky alcohol and drug use, and the timely referral to more intensive substance abuse treatment for those who have substance abuse disorders” [11]. Given that the majority of adolescents (83%) see a physician each year and may be receptive to discussing substance use during these visits, the primary care setting is ideal to offer SBIRT [12]. Screening not only identifies youth currently engaged in alcohol use but also provides positive reinforcement to youth who do not use, a strategy known to delay initiation of alcohol use [13]. However, only 50%-86% of pediatric providers self-report screening for substance use and only a minority of those screen use validated tools [14]. Screening in the absence of a validated tool results in the identification of only 1 in 3 youth with excessive alcohol use [15]. Additionally, providers may be inadequately trained on how to intervene with youth who do report underage drinking, and often communities have few referral options for youth with problematic alcohol use. Therefore, interventions are needed to identify and break down barriers to SBIRT adoption by primary care providers.

### SBIRT

SBIRT is most often used in the primary care setting, but the model can be readily adapted to use in community settings. A recent study of high school nurses found that most (64%) reported screening students with suspected substance use and more than 77% of said nurses favored universal alcohol screening in schools [16]. However, only a minority (18%) of school nurses report using a validated screening tool. The most commonly cited barrier to using a validated model is unfamiliarity with screening tools (36%). The authors concluded that the implementation of SBIRT focused on standardized, annual screening has the potential to deliver high-quality care within the school setting, broadening the reach of substance use screening and prevention beyond the clinical setting.

**Figure 1:**
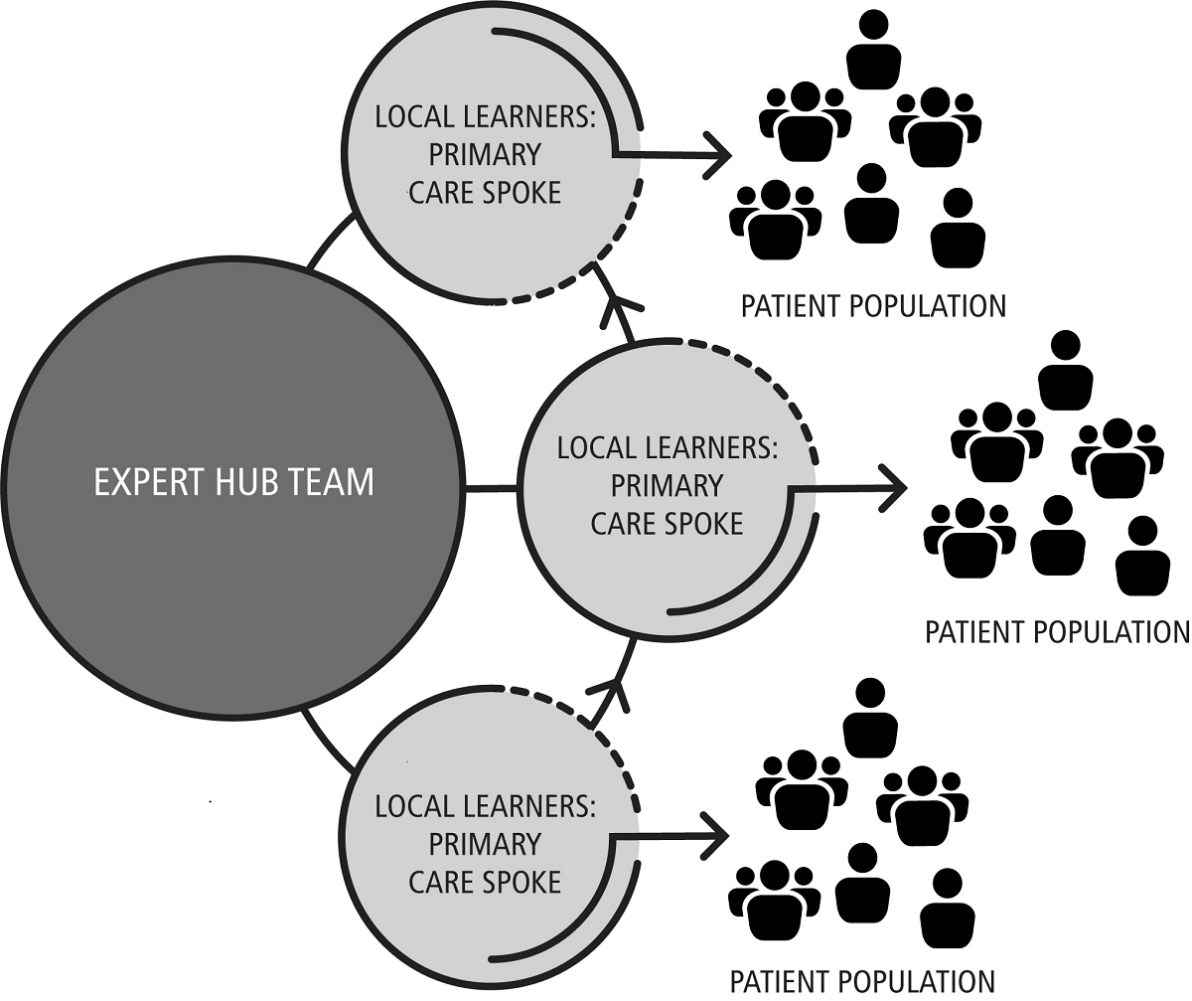
The ECHO model uses a hub and spoke model to create an all-teach, all-learn environment. The more providers that are trained, the more patients that are impacted.

The goal of this protocol is to equip communities with the ability to address underage drinking and opioid use among youth aged 9-20 years through a telehealth platform called Project ECHO (Extension for Community Healthcare Outcomes). Project ECHO was developed at the University of New Mexico and is a model with the power to rapidly transfer knowledge and exponentially increase a community’s capacity to deliver best-practice care to underserved populations through case-based discussion and brief lecture. The ECHO model uses videoconferencing technology as a platform for telementoring and collaborative care, with a hub-and-spoke structure (Figure 1). This protocol aims to train primary care providers and school nurses within a rural 10-county catchment area to use SBIRT and also to facilitate collaboration with community organizations to better coordinate substance use prevention efforts in each county (see Multimedia Appendix 1).

## Methods

The research team will include several professionals who have been involved in the physical and mental care of adolescents, specifically those with substance use and misuse issues. This team will include a pediatrician and adolescent medicine specialist, a pediatrician/internist with addiction certification, a licensed clinical social worker who has managed inpatient adult and pediatric consult-liaison services for three decades, a project manager, a marketing specialist, and a program director. This team of seasoned professionals will identify a number of topics relevant to the identification and screening of substance use disorders by studying motivational and interview-based brief clinical interventions, the biology of alcohol’s effect on the body, risk and protective factors, community-based programming, and the treatment of substance use disorders. There will be a novel curriculum developed specifically for this project, especially as it uses the Project ECHO method to develop and enhance providers’ skills working with substance use in adolescents. To our knowledge, a project of this type is novel and has not been done to date in school settings or pediatricians’ offices; thus, prior curricula were not available.

This protocol will take advantage of three characteristics that make SBIRT an effective behavioral health intervention: (1) brief, validated, universal pre-screening/screening tools; (2) easy to learn intervention approaches that diverse provider types can use; and (3) incorporation of strong referral linkages to specialty treatment. The Project ECHO model will be used to teach SBIRT to school personnel and primary care providers. Further, this protocol includes the creation of a network of experts and participants to support best practices and identify solutions to overcome barriers related to addressing underage alcohol use. This study (STUDY00010821) has been approved by the Pennsylvania State University Institutional Review Board.

### Recruitment

The 10 targeted rural counties in Pennsylvania had an estimated population of 78,833 persons aged 10-19 years in 2017 [17]. Overall, 31%, 46%, and 61% of 8th, 10th, and 12th graders, respectively, reported lifetime alcohol use, compared to 24%, 43%, 56% nationwide, respectively (Table 1) [1]. Likewise, throughout the 10 targeted counties, there was an average of 8.3 alcohol-related crashes per 100,000 crashes. In Pennsylvania as a whole, an average of 4.8 per 100,000 alcohol-related crashes involved youth under 21 years of age. Within the targeted counties, there are 50 school districts and 631 primary care providers (defined as both family medicine and pediatric providers), all being important targets for the proposed project. Within our specific catchment area, school personnel and primary care providers will be recruited through targeted email listservs. Participants must have access to a zoom-capable device and the internet. However, any school personnel or primary care provider in the catchment area will be eligible to participate given that these prerequisites are met.

**Table 1:** Ten rural county catchment area description.

County	Total (N=118,086), n	Lifetime use of alcohol, n (%)			Alcohol-related crashes (per 100,000), n	School districts (n=50), n	Primary care providers (n=631), n
		8th Grade	10th Grade	12th Grade			
							
Blair	16,298	5378 (33)	7823 (48)	9453 (58)	13.6	6	131
Bradford	8060	2902 (36)	4675 (58)	5723 (71)	8.2	6	50
Centre	23,848	8347 (35)	10,493 (44)	15,024 (63)	8.1	5	116
Franklin	21,300	4473 (21)	4686 (22)	7668 (36)	12.3	5	124
Fulton	1915	632 (33)	1245 (65)	1379 (72)	6.8	4	4
Northumberland	11,156	3904 (35)	6024 (54)	7698 (69)	2.2	5	39
Perry	6056	2301 (38)	2967 (49)	3694 (61)	10.9	4	16
Schuylkill	17,675	6717 (38)	10,075 (57)	13,080 (74)	4.2	10	101
Snyder	6299	1827 (29)	3150 50)	4283 (68)	2.5	2	24
Tioga	5479	438 (8)	877 (16)	1918 (35)	14.5	3	26

### Proposed Approach

**Figure 2:**
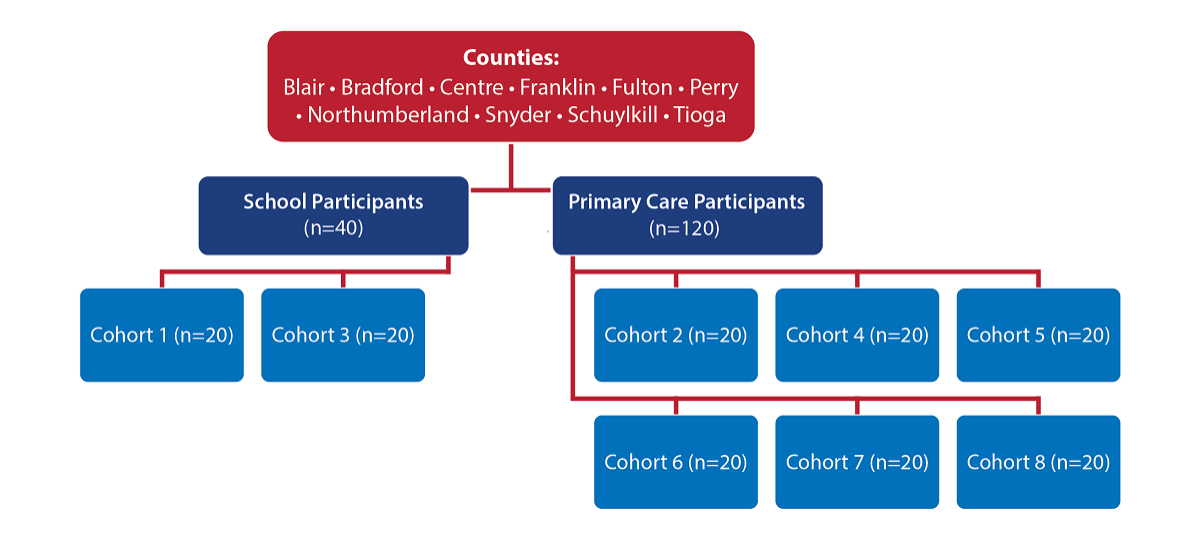
Recruitment into ECHO Cohorts.

This protocol includes the implementation of an SBIRT training model for primary care clinics and schools to prevent the onset and reduce the progression of substance abuse, reduce substance abuse-related problems, and strengthen prevention infrastructure at the community level (Table 2). Specifically, we will recruit and train 120 primary care providers and allied health professionals and 20 school nurses to participate in SBIRT training (Figure 2). We will use a research-based SBIRT model which includes six characteristics noted by SAMHSA: (1) initial screening is brief; (2) screening is universal; (3) specific behaviors will be targeted; (4) services occur in a public health setting (ie, primary care clinics and schools); (5) program is comprehensive; and (6) model is supported by strong research and experiential evidence [11]. Lastly, project feedback will be obtained annually from local Communities That Care (CTC) coalitions.

**Table 2:** Proposed goals and measurable objectives.

Goals	Measurable objectives
Increase the capacity of primary care providers to provide SBIRT^a^ to youth aged 9-20 years to reduce alcohol and opioid use and provide treatment or referral for those currently using these substances.	By the end of 2024, our team will have trained 20% of primary care providers in the 10-county catchment through Project ECHO^b^ (n=120). This enables approximately 57,600 youth to be served by SBIRT over the proposed project timeline.
Increase the capacity of schools to provide SBIRT to youth aged 9-20 years to reduce alcohol use and opioid use and provide treatment and, if necessary, referral for those currently using these substances.	By the end of 2024, our team will have trained at least 20 school nurses within the 10-county catchment through Project ECHO.
Increase alcohol and substance use prevention resources and programs for primary care clinics and schools to distribute to youth and families.	By the end of 2024, at least 50% of school buildings and primary care clinics will have alcohol/substance use prevention toolkits available for youth and families.

^a^SBIRT: screening, brief intervention, and referral to treatment.

^b^ECHO: Extension for Community Healthcare Outcomes.

Further, this study will follow the SAMHSA-recommended multi-stage screening approach. Clinical staff will prescreen and if a patient prescreens positive, a full screen will be conducted. Low-risk patients will be supported for their health decisions. Moderate-risk patients will receive a brief intervention. Patients with high or severe risk/dependency will be referred for specialty treatment. Referral requires primary care clinics and schools to establish links to appropriate services, which can be a significant barrier. We will reduce this barrier by providing linkages through existing CTC networks that are located throughout communities in Pennsylvania. The 10 counties included in this project also have coalitions in place, some of which will be CTCs supported by the Pennsylvania Commission on Crime and Delinquency. CTC is an evidence-based approach that takes communities through a well-defined and structured process to prevent adolescent problem behaviors and promote positive youth development.

**Figure 3:**
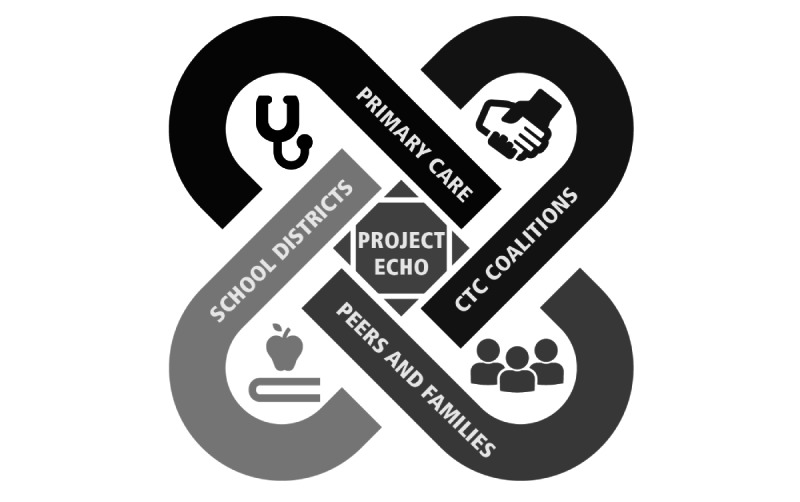
This is a collaboration between primary care providers and school districts, with input from communities that care coalitions and outreach to peers and families.

The SBIRT training curriculum will use the approach of the American Academy of Pediatrics’ 2016 clinical report [14]. This simplified, clinical approach will support successful research-informed SBIRT implementation across our 10 rural counties. By working with the networks created for primary care physicians and school nurses, we will identify and implement a comprehensive prevention approach (SBIRT) within these settings to include a mix of evidence-based programs, policies, and practices that best address alcohol and opioid use (Figure 3). Additionally, families and peers will have access to fact sheets and handouts that promote health behaviors.

### Project ECHO

The ECHO model has four core principles: (1) use technology to leverage scarce resources; (2) share best practices to reduce disparities; (3) employ case-based learning to master complexity; and (4) monitor outcomes to ensure benefit. Our program will use Project ECHO to train primary care providers and school nurses in our 10-county catchment area to provide SBIRT intervention to reduce rates of underage drinking and opioid use among youth aged 9-20 years. The ECHO model uses videoconferencing technology as a platform for telementoring and collaborative care, with a hub-and-spoke structure (Figure 1). Community-based providers (the spokes) will participate in weekly virtual ECHO sessions with a multidisciplinary specialty team at Penn State College of Medicine (the hub) who will provide technical assistance and training on the use of SBIRT and will facilitate discussions of cases brought forward by providers. These experts include a pediatrician, a board-certified Addiction Medicine specialist who is also a pediatrician, and a social worker. Providers share knowledge, expertise, and experience. Over time, providers become experts, engaged in a wider community of learners and empowered to address substance use in their patients. They have the support of specialists for advice and referral, creating an effective triage system and direct linkage to care.

Through case-based discussions involving all spoke participants and brief lectures provided by experts on the hub, the ECHO model will create a network of learners with demonstrated effectiveness at improving competence, performance, knowledge, attitudes, and confidence in treatment related to substance use disorders [12]. To incentivize participation, continuing education credits will be awarded for attending each session.

### Curriculum

Two overlapping curricula have been developed, one for school personnel, tailored to fit the school setting, and a second for pediatricians in the clinical setting. The curriculum includes the topics listed in Table 3. Unlike SBIRT for adults, these sessions will take into account the unique phase of adolescence, including transitions, peer pressure, adverse childhood experiences, and other factors that may impact an adolescent's likelihood to consume alcohol or use substances. We will develop 2 separate curricula in order to address the specific needs of school personnel (nurses, social workers, and guidance counselors) compared with those of primary care pediatricians. Although most of the topics covered will be provided to both groups, the curriculum for the physicians will contain specific information about the formal diagnosis of substance use disorders in adolescents, medical treatment available for substance use disorders, and billing for screening for and treatment of substance use issues.  For school personnel, the curriculum will focus on several topics different from the pediatrician group such as recognizing and identifying substance use in school settings, triaging substance use concerns, and the risk and protective factors that are inherent in school settings (eg, school failure, poor school performance, school connections, and support).

**Table 3:** Proposed curriculum topics.

Curriculum topics	School personnel	Pediatricians
Introduction to SBIRT^a^ and brief interventions	✓	✓
Operationalizing screenings and billing for services		✓
Addressing vulnerability during life transitions	✓	✓
Identifying substance use in adolescents	✓	
Diagnosing substance use in adolescents		✓
Beyond alcohol: substance use prevention	✓	✓
Adolescent progress of substance use	✓	✓
How alcohol affects human biology	✓	✓
Referral to psychosocial and medication treatment	✓	✓
Adolescent risk factors vs protective factors	✓	
Medication for substance use disorders		✓
Positive development: building good habits	✓	✓
Review of brief interventions and introduction to communities that care	✓	✓

^a^SBIRT: screening, brief intervention, and referral to treatment.

### Proposed Evaluation

We have developed a tracking database within REDCap that will enable us to collect and analyze data from all participating providers and school nurses. The ECHO model aims to evaluate outcomes within Moore’s Seven Levels for CME Outcomes Measurements [18]. This progressive approach to evaluation is the recommended framework for evaluating Project ECHO from its founders at the University of New Mexico. Our current database collects baseline and post-program knowledge, skills, attitudes, and confidence, as well as weekly questionnaires that assess provider engagement, intent to make changes in practice, increased ability to provide appropriate care, and decreased professional isolation. REDCap is an encrypted, HIPAA (The Health Insurance Portability and Accountability Act of 1996)-compliant web-based application designed to support data capture for research studies. REDCap access will require user authentication with a password and limits data access based on an individual’s role in a project. Further, the study team will access county-level data from a variety of publicly available sources (Multimedia Appendix 2).

### Data Analysis

Researchers will compare the outcome measures by treatment type (eg, initiation of buprenorphine/naloxone in the emergency department and type of medication assisted treatment) and referral source using appropriate epidemiologic study designs and statistical methods (eg, a quasi-experiment or an ecologic study design to evaluate the program impact at the community level, chi-square tests for categorical variables or t-tests for continuous variables, or the multivariable generalized linear model, etc). This analysis will allow us to better understand larger trends that occur over the proposed project period.

### Community Engagement

By linking CTC partners with primary care providers and school personnel, we aim to strengthen the prevention system and ensure sustainability. This protocol aims to share evaluation data collected from participating providers and school nurses with the CTC’s to promote data-driven decision making by the CTCs. The CTCs will then be better able to connect schools and clinics with evidence-based resources in the community, address technical assistance and training needs of providers and schools, and use existing services more efficiently. Further, we will develop effective prevention messages and other prevention strategies for use by the schools and clinical practices within our catchment area. In addition, the study team will make educational and evidence-based resources available to school nurses, pediatricians, or other practice group personnel for distribution to youth and caregivers who are seeking care. Resources will include patient/family health questionnaires and assessments, protocols for parents to follow, fact sheets for practitioners, parents and youth, and handouts for parents to assist them with promoting healthy behaviors.

## Results

Our study launched 1 SBIRT ECHO with 34 school personnel in March 2020. School participants from our first ECHO series are from both rural (n=17) and urban (n=17) counties, although 12 of the urban school districts fall within counties that contain rural school districts. Participants include school nurses (n=15), school counselors (n=8), teachers (n=5), administrators (n=3), and social workers (n=3). Before the study began, just 2 of 13 (15%) of these schools indicated that they were screening for alcohol use. The next steps include launching our first ECHO series with pediatricians and primary care providers in the fall of 2020.

## Discussion

### Overview of Proposed Findings

The overarching goal of this research is to decrease adolescent alcohol consumption and opioid usage by increasing the use of screening, brief interventions, and referrals to treatment by both school personnel and primary care providers. Because adolescents in rural areas are at higher risk for alcohol and substance use, targeting rural counties with supportive community components is important to curbing adolescent alcohol consumption and resultant negative health outcomes. Using publicly available data such as the Pennsylvania Youth Survey data, SAP referrals, and alcohol-related crashes in underage drivers will allow insight into whether or not this approach is effective [1].

### Participants

Prior studies point to the need for interdisciplinary teams in schools to work towards improved health services for students [19]. Although this protocol originally aimed to recruit school nurses, a diverse group of school personnel joined the study including social workers, physical education teachers, health education teachers, and guidance counselors. We anticipate that a group of interdisciplinary participants will further enhance discussion and prove to be more beneficial for participants. Engaging pediatricians and primary care providers is also critical given the underutilization of substance use screening and intervention in this frontline health care setting.

### Limitations of Research Design

Although this study does not evaluate student perceptions of SBIRT, prior studies have shown that students who screen negatively for substance use view routine screening favorably. Students that report having used illicit substances view drug and alcohol screenings less favorably [20].

Although this study entails rigorous data collection, several other programs exist in the counties of intervention. Therefore, there is a strong possibility of residual confounding. It will be difficult to determine if successes should be attributed to Project ECHO or other programming in the community.

Because this study is targeting rural communities that have strong community coalitions and resources to help families and adolescents that screen positive for alcohol or substance use, results may be less transferrable to communities that do not have strong coalitions already in place. Additionally, as this study targets a rural catchment area, the results may not be transferrable to urban counties.

Lastly, having a study span of 5 years increases the likelihood of changes in existing data collection measures. For example, if schools stop collecting 30-day alcohol and opioid usage through the Pennsylvania Youth Survey, it will be very difficult to measure adolescent alcohol and substance use.

### Study Potential

Currently, the US Preventive Services Task Force’s recommendations for children aged 12-17 years is “that the current evidence is insufficient to assess the balance of benefits and harms of screening and brief behavioral counseling interventions for alcohol use in primary care settings” [21]. The task force has similar guidelines related to illicit drug use in that “the current evidence is insufficient to assess the balance of benefits and harms of primary care-based behavioral counseling interventions to prevent illicit drug use, including nonmedical use of prescription drugs, in children, adolescents, and young adults ” [22]. Therefore, this study aims to help close the gap in evidence to help move screening, brief interventions and referrals forward to get adolescents the help that they need. The evidence for SBIRT is limited in regards to adolescents. However, prior studies have evaluated Project ECHO in regards to training primary care physicians to address the opioid epidemic. One study in Ontario, Canada found that participation in Project ECHO increased provider knowledge regarding opioids and pain management [23]. Another study from Pennsylvania indicated that 6 months of participation in Project ECHO resulted in significantly decreased wait times for patients seeking treatment for opioid use disorders [24]. These studies demonstrated improved physician knowledge, which enabled primary care providers to optimize care for patients with opioid use disorders.

Although not all states have large rural areas and limited access to care, this study applies to at least 18 other states that have a similar percentage of rural area within their borders [16]. A comprehensive SBIRT model has the potential to address alcohol and other substance use outside of clinical settings, which may be particularly important within rural communities. In this way, engaging and training school nurses from the 10-county catchment area will further increase county capacity to implement, sustain, and improve effective substance abuse prevention services.

### Conclusion

Comprehensive substance use disorder prevention involves individual, environmental, and collaborative strategies across schools, homes, communities, and health-focused regulatory bodies. A multipronged approach is the key to changing social norms and expectations for substance use.

SBIRT has been defined by SAMHSA as “a comprehensive integrated public health approach to the delivery of intervention for individuals with risky alcohol and drug use, and the timely referral to more intensive substance abuse treatment for those who have substance abuse disorders” [10]. This project plans to implement a comprehensive SBIRT education model in primary care clinics and schools to prevent the onset and reduce the progression of substance abuse, reduce substance abuse-related problems, and strengthen prevention capacity and infrastructure at the community level. Specifically, we will recruit 120 primary care providers and allied health professionals as well as 20 school nurses to participate in SBIRT training. This is an innovative model to improve rural adolescent health by reducing alcohol and opioid-related harms. The data that support the findings of this study will be available on request from the corresponding author. The data will not be publicly available due to privacy or ethical restrictions.

## Appendix

Multimedia Appendix 1 This will be a 5-year project including IRB approval, data evaluation, 10 ECHO cohorts, annual community advisory board meetings, and outreach strategies including the dissemination of marketing materials.

Multimedia Appendix 2 Performance measures will be taken from a wide array of project surveys and public databases.

Multimedia Appendix 3 Peer Review Comments.

## Abbreviations

CTC: Communities That Care
ECHO: Extension for Community Healthcare Outcomes
SAMHSA: Substance Abuse and Mental Health Services Administration
SBIRT: Screening, brief intervention, and referral to treatment
