# Supplementary material for: Reducing Alcohol and Opioid Use Among Youth in Rural Counties: An Innovative Training Protocol for Primary Health Care Providers and School Personnel
Source: JMIR Res Protoc. 2020 Nov 6;9(11):e21015. doi: 10.2196/21015 (PMC7679207; doi:10.2196/21015)
Supplement: Multimedia Appendix 1 [file resprot_v9i11e21015_app1.docx]

**Multimedia Appendix 1: Project Timeline**

| **Project Required Activities**  **(Responsible Staff)** | **Year 1** | | | | **Year 2** | | | | **Year 3** | | | | **Year 4** | | | | **Year 5** | | | |
| --- | --- | --- | --- | --- | --- | --- | --- | --- | --- | --- | --- | --- | --- | --- | --- | --- | --- | --- | --- | --- |
| Obtain IRB approval | x | x |  |  |  |  |  |  |  |  |  |  |  |  |  |  |  |  |  |  |
| Project ECHO cohort 1-2 – school nurses | x | x | x | x |  |  |  |  |  |  |  |  |  |  |  |  |  |  |  |  |
| Evaluation design and procedures, data collection/security, and project reporting | x | x | x | x | x | x | x | x | x | x | x | x | x | x | x | x | x | x | x | x |
| Community Advisory Board meeting |  |  |  | x |  |  |  | x |  |  |  | x |  |  |  | x |  |  |  | x |
| Project ECHO cohorts 3-8 – primary care providers |  |  | x | x | x | x | x | x | x | x | x | x | x | x | x | x | x | x | x | x |
| Develop outreach strategies |  |  |  | x | x | x | x | x | x | x | x | x | x | x | x | x | x | x | x | x |
| Development/dissemination of Marketing materials |  |  |  |  | x | x | x | x | x | x | x | x | x | x | x | x | x | x | x | x |
