# Supplementary material for: Reducing Alcohol and Opioid Use Among Youth in Rural Counties: An Innovative Training Protocol for Primary Health Care Providers and School Personnel
Source: JMIR Res Protoc. 2020 Nov 6;9(11):e21015. doi: 10.2196/21015 (PMC7679207; doi:10.2196/21015)
Supplement: Multimedia Appendix 2 [file resprot_v9i11e21015_app2.docx]

**Multimedia Appendix 2: Performance Measures**

| **Performance Measure** | **Data Source** | **Collection Frequency** |
| --- | --- | --- |
| **Project-level Measures** | | |
| Number of participants trained in SBIRT | ECHO clinic evaluation | Monthly |
| Number of participants that implemented SBIRT in practice | ECHO clinic 6 month follow up survey | Annually |
| Number of youths reached by SBIRT | Schools, public record, interviews | Biannually |
| Number of CtC Evidence-based programs  shared | Program records | Monthly |
| Number of new Evidence-based programs implemented in Schools | Program records | Monthly |
| Number of marketing materials disseminated | Program records | Monthly |
| Provider knowledge, confidence & skills | ECHO clinic evaluation | Biannually |
| CtC Board feedback | Focus group or interviews | Annually |
| Patient perception of care | Focus groups | Annually |
| **National Outcome Measures** | | |
| ***Abstinence*** | | |
| Abstinence from alcohol use | PAYS data | Every 2 years |
| ***Resilience*** | | |
| Referrals to Student Assistance Program | School building data | Annually |
| Referrals to Treatment | Survey with clinic administration^17^ | Quarterly |
| School dropout rates | School records, U.S. Census (American Community Survey) | Pre/Post |
| Alcohol-related crashes in underage drivers | Penn DOT and, CDC’s WISQARS™ (Web-based Injury Statistics Query and Reporting System) | Annually |
| ***Criminal Justice Status*** | | |
| Juvenile delinquency | Kids Count Data Center | Annually |
| Arrest rates for liquor law violations | US Bureau of Justice Statistics and local police records, US Census (American Community Survey) | Annually |
| Arrest rates for Drunkenness and DUI | US Bureau of Justice Statistics and local police records, US Census (American Community Survey) | Annually |
