# Supplementary material for: Reducing Alcohol and Opioid Use Among Youth in Rural Counties: An Innovative Training Protocol for Primary Health Care Providers and School Personnel
Source: JMIR Res Protoc. 2020 Nov 6;9(11):e21015. doi: 10.2196/21015 (PMC7679207; doi:10.2196/21015)
Supplement: Multimedia Appendix 3 [file resprot_v9i11e21015_app3.pdf]

SUMMARY STATEMENT
(Privileged Communication)

		Application Number:	**1 H79 SP081725-01**
Review Group:	**1904-P**	*Requested Start Date:*	**09/30/2019**
IRG Meeting Dates:		*Anticipated Council:*	**AUG 2019**
		Notice Number:	**SP19-004**
		Notice Title:	**Strategic Prevention Framework - Partnerships for Success**

PENNSYLVANIA STATE UNIV HERSHEY MED CTR
Johnson, Stephanie A
Director, Grants Administration
PENNSYLVANIA STATE UNIV HERSHEY MED CTR
500 University Drive
P.O. Box 850, Mail Code H138
Hershey, PA 17033

Project Title:	**Collaborative Dissemination of SBIRT Training and Prevention Resources to Address Alcohol Use in Youth within Rural Pennsylvania**
IRG Action:	**Scored(SC)** *Priority Score:* **91**
**Participant Protection:**	**34 No IRG Comments or Concerns**

FY 2019 Strategic Prevention Framework – Partnerships for Success (SPF-PFS)

SP-19-004

Application Abstract

One in three Pennsylvanian eighth graders has used alcohol, significantly higher than the national average (23%). We propose addressing underage drinking in ten rural counties in Central PA through training primary care and school personnel in Screening, Brief Intervention, Referral, and Treatment (SBIRT) using telehealth, specifically Project ECHO. Importantly, these counties also have in place Communities that Care (CtC) coalitions, an evidence-based approach that takes communities through a well-defined and structured process to prevent adolescent problem behaviors and promote positive youth development. By identifying CtC partner coalitions for the proposed project, we effectively integrate the SAMHSA Strategic Prevention Framework (SPF).

Rural communities are particularly critical to address given that alcohol use by rural youths is higher than use by their urban counterparts (37.8% vs. 34.3%). Overall, 31%, 46%, and 61% report alcohol use in 8th, 10th, and 12th grades, which is higher than the national average (24%, 43%, 56%, respectively). Our ten selected rural counties have an estimated 2017 population of 78,833 persons aged 10-19 years. Within these counties there are 50 school districts and 631 primary care providers (defined as both family medicine and pediatric providers), important targets for our proposed intervention. One service gap for addressing underage drinking is the lack of appropriate screening, intervention and referral for alcohol use among youth. Guidelines recommend primary care providers incorporate screening, brief intervention, and referral to treatment (SBIRT) into routine care. Engaging and training primary care providers and school nurses from our 10-county rural catchment area in SBIRT will enhance the infrastructure to increase the capacity to implement, sustain and improve effective substance abuse prevention services. Specifically, our goals include increasing the capacity of primary care providers and school nurses to provide SBIRT to persons ages 9-20 to reduce alcohol use and opioid use and provide treatment and/or referral for those currently using these substances. By the end of 2024, our team will have trained 20% of primary care providers and 20 school nurses within the ten-county catchment area through Project ECHO. As a result, we anticipate benefiting more than 42,000 youth aged 9-20 over the proposed project period.

Priority Score
91

Review Resume

This application was reviewed by a SAMHSA Peer Review Committee to provide an objective assessment of the merit of the applicant organization's response to the Evaluation Criteria published in the funding announcement (FOA).

The Section scores will have the following verbal descriptors:

Outstanding = All criteria are thoroughly addressed, strongly developed, and well supported. Documentation and required information are specific and comprehensive. Application is extremely strong with insignificant weaknesses. Weaknesses identified will likely have no impact on the successful implementation of the proposed project.

Very good = Criteria are thoroughly addressed with necessary detail and clearly supported. Documentation and required information are specific and feasible. Application is very strong with only some minor weaknesses. Weaknesses identified will likely have minor impact on the successful implementation of the proposed project.

Acceptable = Criteria are addressed, but do not contain necessary detail and/or support. Most documentation and required information are present and sufficient, although some are deficient or missing. Application has some strengths but with at least one major weakness. Weaknesses identified will likely have moderate impact on the successful implementation of proposed project.

Marginal = Some criteria are addressed, although when addressed, do not contain necessary detail and/or support. Some documentation and required information are missing or deficient. Application has a few strengths and a few major weaknesses. Weaknesses identified will likely impact the successful implementation of the proposed project.

Unacceptable = Few, if any, criteria are addressed. Documentation and required information are missing. Application has very few strengths and numerous major weaknesses. Weaknesses identified will likely prevent the successful implementation of the proposed project.

OR

The criteria do not meet the programmatic intent of the Funding Opportunity Announcement.

Section A: Statement of Need

<u>Maximum points = 15</u>

In this Section of the Project Narrative, the applicant organization must identify where the project will be implemented and the population(s) that will be impacted by the infrastructure development in the targeted systems or agencies. The applicant organization must document the need for enhanced infrastructure to increase the capacity to implement, sustain, and improve effective substance abuse prevention services in the proposed catchment area that is consistent with the purpose of the FOA. It must identify any substances other than alcohol that it will address in the project. Furthermore, it must describe the extent of the problem in the catchment area, including service gaps, and document the extent of the need (i.e., current prevalence rates or incidence data) for the population(s) of focus identified.

Overall Assessment of Section A: Population of Focus and Statement of Need

**Outstanding (15-14)**		**Very Good (13-12)**		**Acceptable (11)**		**Marginal (10-9)**		**Unacceptable (8-0)**
**X**								

Strengths:

<u>X</u> The narrative clearly identifies:

  <u>X</u> the population(s) that will be impacted by the infrastructure development in the targeted systems or agencies

  <u>X</u> the proposed catchment areas where the project will be implemented

<u>X</u> Extensive documentation is provided on the need for an enhanced infrastructure to increase the capacity to implement, sustain, and improve effective substance abuse prevention services in the proposed catchment areas that is consistent with the purpose of the FOA.

<u>X</u> If applicable, identifies any substances other than alcohol that it will address in the project.

<u>X</u> The service gaps and other problems related to the need for infrastructure development are thoroughly discussed.

<u>X</u> Data sources are identified.

<u>Additional comments:</u> None.

Weaknesses:

☐The narrative does not clearly identify:

  ☐the population(s) that will be impacted by the infrastructure development in the targeted systems or agencies

  ☐the proposed catchment areas where the project will be implemented

☐The population of focus does not align with the intent of the FOA.

☐Substantive documentation is not provided on the need for an enhanced infrastructure to increase capacity to implement, sustain, and improve effective substance abuse prevention services in the proposed catchment area that is consistent with the purpose of the FOA.

☐If applicable, substances other than alcohol that will be addressed in the project are not identified.

□The service gaps and other problems related to the need for infrastructure development are not thoroughly discussed
□Does not identify all data sources.

Additional comments: No significant weaknesses noted.

Section B: Proposed Implementation Approach

Maximum points = 35

In this Section of the Project Narrative, the applicant organization must describe the goals and measurable objectives of the proposed project and align them with the Statement of Need outlined in A.2. It must also state the unduplicated number of individuals it proposes to serve (annually and over the entire project period) with grant funds. In addition, the applicant organization must describe how it will implement the Required Activities and provide a chart or graph depicting a realistic timeline for the entire five years of the project period showing dates, key activities, and responsible staff.

Overall Assessment of Section B: Proposed Implementation Approach

Outstanding (35-32)		Very Good (31-28)		Acceptable (27-25)		Marginal (24-21)		Unacceptable (20-0)
		X						

Strengths:
X The narrative clearly describes:
X the goals and objectives of the proposed project
X how the proposed goals and objectives will align with the applicant organization's Statement of Need.
X Measureable objectives are provided which will allow for the success of the project to be evaluated.
X A comprehensive description is provided of how the Required Activities will be implemented.
□A chart or graph depicting a realistic timeline is provided which includes dates, key activities, and responsible staff for the entire 5 year project period.

Additional comments: None.

Weaknesses:
□The narrative does not clearly describe:
□the goals and objectives of the proposed project
□how the proposed goals and objectives will align with the applicant organization's Statement of Need
□All of the objectives are not measurable.
□A comprehensive description is not provided of how the Required Activities will be implemented.

☐A chart or graph depicting a realistic timeline is not provided.
X A chart or graph depicting a realistic timeline is provided but omits the required elements:
- ☐describes a five-year project period
- ☐states key dates, which show project implementation and service delivery can begin as soon as possible and no later than four months after, grant award
- X key activities that support infrastructure development
- X responsible key staff that include a Project Director and Lead Epidemiologist

Additional comments: None.

Section C: Staff, Management and Relevant Experience

Maximum points = 20

In this Section of the Project Narrative, the applicant organization must describe the experience of its organization with similar projects and providing services to the population(s) of focus for this FOA. It must also identify any other organization(s) that will partner in the proposed project. The applicant organization must provide a complete list of staff positions for the project, including the Key Personnel (Project Director and Lead Epidemiologist) and other significant staff members. In addition, it must also describe the role of each, their level of effort, and qualifications, including their experience providing services to the population(s) of focus and familiarity with their culture(s) and language(s).

Overall Assessment of Section C: Staff, Management and Relevant Experience

Outstanding (20-18)		Very Good (17-16)		Acceptable (15-14)		Marginal (13-12)		Unacceptable (11-0)
		X						

Strengths:
X Thoroughly describes the experience of its organization with similar projects and providing services to the population(s) of focus for this FOA
X The applicant organization clearly identifies if it will partner with any other organization(s) in the proposed project
X A complete list of staff positions, including Key Personnel (Project Director and Lead Epidemiologist) for this project is provided and includes:
- X description of roles

X level of effort
☐qualifications
☐description of experience providing services to the population(s) of focus
☐familiarity with their culture(s) and language(s)

Additional comments: None.

Weaknesses:
☐Specific information is not provided about the applicant organization’s experience, roles, and responsibilities with similar projects and/or providing services to the population(s) of focus.

□The applicant organization does not clearly identify if it will partner with other organization(s) in the proposed project
□A complete list of staff positions, including Key Personnel (Project Director and Lead Epidemiologist), for this project is not provided.
□ The Key Personnel (Project Director and Lead Epidemiologist) is not identified in the list of staff positions.
□ It is not clear that the level of effort for some of the key staff will be sufficient to fulfill their assigned responsibilities.
□A complete list of staff positions, including Key Personnel (Project Director and Lead Epidemiologist), for this project is provided but omits the following:
□description of roles
□level of effort
X qualifications
X description of experience providing services to the population(s) of focus
X familiarity with their culture(s) and language(s).

Additional comments: none.

Section D: Data Collection and Performance Assessment

Maximum points= 30

In this Section of the Project Narrative, the applicant organization must provide specific information about how it will collect the required data for this program and how such data will be utilized to manage, monitor and enhance the program.

Overall Assessment of Section D: Data Collection and Performance Assessment

Outstanding (30-27)		Very Good (26-24)		Acceptable (23-21)		Marginal (20-18)		Unacceptable (17-0)
X								

Strengths:
X Detailed information is provided about how the required data for the program will be collected.
X Clear documentation is provided about how data will be used to manage the program.
X A thorough description is provided about how data will be used to monitor the program.
X Specific information is provided about how data will be used to enhance the program.

Additional comments: None.

Weaknesses:
□ Detailed information is not provided about how the required data for the program will be collected.

 Insufficient documentation is provided about how data will be used to manage the program.
 A thorough description is not provided about how data will be used to monitor the program.
 Specific information is not provided about how data will be used to enhance the program.

Additional comments: No significant weaknesses noted.

Participant Protection Overview

Confidentiality and Participant Protection

The Committee reviewed the applicant organization's plans for ensuring confidentiality and SAMHSA participant protection and found them satisfactory.
